# 0968. The influence of hypothermia and catecholamines on guinea pig's small bowel motility *in vitro*

**DOI:** 10.1186/2197-425X-2-S1-P67

**Published:** 2014-09-26

**Authors:** M Schörghuber, E Tatzl, P Holzer, W Toller, S Fruhwald

**Affiliations:** Department of Anesthesiology and Intensive Care M, Medical University of Graz, Graz, Austria; Institute of Experimental and Clinical Pharmacology, Medical University of Graz, Graz, Austria

## Introduction

In critically ill patients early enteral nutrition (EN) preserves gastrointestinal (GI) integrity and motility and should be started as early as possible. We know that several therapeutic strategies, e.g. catecholamines or analgosedation, exert adverse effects on GI motility.^1^ What we do not know is whether therapeutic hypothermia has an influence on GI motility and thereby feeding intolerance.

## Objectives

The aim of this study was to find out if guinea pig's small bowel motility is altered during hypothermia and after rewarming and if catecholamines cause alterations of peristalsis in this situation.

## Methods

Guinea pig´s small bowel segments of 8 cm length were set up in organ baths containing oxygenated Tyrode´s solution. Peristalsis was elicited by luminal perfusion (0.5 ml/min) against an aboral resistance of 400 Pascal (Pa). Perfusion of the segments resulted in an increase of the intraluminal pressure up to a pressure threshold (PT; mean ±SEM), where peristaltic contractions were triggered. The pressure was recorded at the aboral end of the segments. An increase of the PT indicates an inhibition of peristalsis, while a decrease of the PT represents a stimulation of peristalsis. A PT of 400 Pa was equated with a complete block of peristalsis. PT was firstly measured at 37°C temperature of the organ bath, after rapid cooling to 20°C and after rewarming to 37°C (control). In a second setting before rewarming one of the following substances were added to the organ baths: adrenaline 100 nM, dobutamine 100 µM, noradrenaline 1 µM. At 37°C PT was evaluated again.

## Results

**Figure 1:**
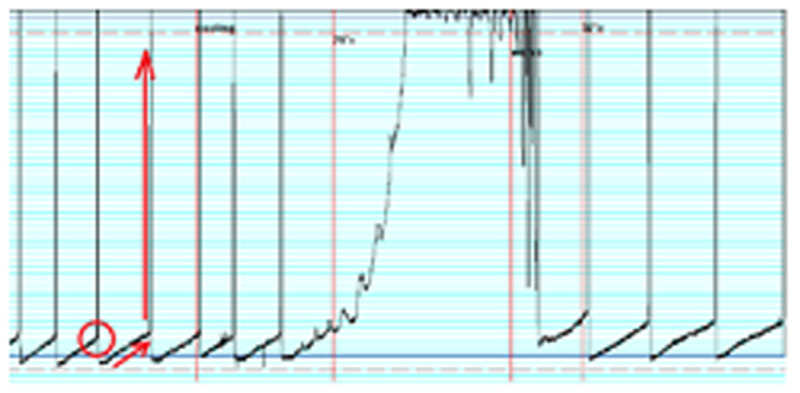
Alteration of peristalsis during hypothermia → incease of intraluminal pressure. O PT. ↑ peristaltic reflex

Basic PT was 49.6 ±6.5 Pa. Lowering the bath temperature to 20°C led to a complete block of peristalsis in all tested segments (PT= 400 Pa, figure 1). During rewarming all small bowel segments started peristaltic contractions spontaneously and showed normal peristalsis at 37°C. In the second setting additional catecholamines resulted in a significantly delayed restart of peristalsis after rewarming and a persistent inhibition of peristalsis (i.e. higher PT) compared to control segments.

**Table 1:** PT and start of peristalsis after admission of catecho/amines compared to control. ^1^two-sided t-test. ^2^Kruskal-Wallis.

			Start of peristalsis after rewarming (%)			
	**PT (Pa) after rewarming**	**p** ^**1**^	Immediately	<5 minutes	>5 minutes	**P** ^**2**^
Control	70.4 ± 8.3					
Adrenaline	182.7 ± 39.3	0.019	10	60	30	<0.001
Dobutamine	264.2 ± 46.6	0.001	0	55	45	<0.001
Noradrenaline	223.9 ± 48.4	0.008	0	40	55	<0.001

## Conclusions

Our experimental setting demonstrates a distinct impairment of small bowel motility during hypothermia, a delayed restart and a persistent inhibition of motility in the presence of catecholamines, explaining the higher incidence of feeding intolerance in this group of patients.
